# Lead Discovery of Type II BRAF V600E Inhibitors Targeting the Structurally Validated DFG-Out Conformation Based upon Selected Fragments

**DOI:** 10.3390/molecules21070879

**Published:** 2016-07-16

**Authors:** Qingwen Zhang, Xuejin Zhang, Qidong You

**Affiliations:** 1 Division of Medicinal Chemistry, Shanghai Institute of Pharmaceutical Industry, 285 Gebaini Road, Pudong, Shanghai 201203, China; 2 State Key Laboratory of Lead Compound Research, WuXi AppTec, 288 Fute Zhong Road, Waigaoqiao Pilot Free Trade Zone, Pudong, Shanghai 200131, China; cherryjin1031@gmail.com; 3 Department of Medicinal Chemistry, School of Pharmacy, China Pharmaceutical University, 24 Tongjia Xiang, Gulou, Nanjing 210009, Jiangsu, China; youqd@163.com

**Keywords:** fragment-based lead discovery, type II kinase Inhibitor, privileged fragments, classical DFG-out conformation, BRAF V600E

## Abstract

The success of the first approved kinase inhibitor imatinib has spurred great interest in the development of type II inhibitors targeting the inactive DFG-out conformation, wherein the Phe of the DFG motif at the start of the activation loop points into the ATP binding site. Nevertheless, kinase inhibitors launched so far are heavily biased toward type I inhibitors targeting the active DFG-in conformation, wherein the Phe of the DFG motif flips by approximately 180° relative to the inactive conformation, resulting in Phe and Asp swapping their positions. Data recently obtained with structurally validated type II inhibitors supported the conclusion that type II inhibitors are more selective than type I inhibitors. In our type II BRAF V600E inhibitor lead discovery effort, we identified phenylaminopyrimidine (PAP) and unsymmetrically disubstituted urea as two fragments that are frequently presented in FDA-approved protein kinase inhibitors. We therefore defined PAP and unsymmetrically disubstituted urea as privileged fragments for kinase drug discovery. A pharmacophore for type II inhibitors, 4-phenylaminopyrimidine urea (4-PAPU), was assembled based upon these privileged fragments. Lead compound **SI-046** with BRAF V600E inhibitory activity comparable to the template compound sorafenib was in turn obtained through preliminary structure–activity relationship (SAR) study. Molecular docking suggested that **SI-046** is a bona fide type II kinase inhibitor binding to the structurally validated “classical DFG-out” conformation of BRAF V600E. Our privileged fragments-based approach was shown to efficiently deliver a bona fide type II kinase inhibitor lead. In essence, the theme of this article is to showcase the strategy and rationale of our approach.

## 1. Introduction

The protein kinase complement of the human genome, the human kinome, encodes about 518 protein kinases which play pivotal roles in virtually all aspects of cellular processes [1,2]. Dysregulation of protein kinase function has been implicated in many human diseases such as cancer, and inflammatory and autoimmune diseases [3]. So far, the US FDA has approved 30 small molecule protein kinase inhibitors, mainly for cancer indications [4].

Protein kinase inhibitors have been generally categorized into four classes—type I, type II, type III, and type IV—based upon their binding mode [5,6,7,8]. Type I inhibitors bind to the active DFG-in conformation, wherein the Asp of the DFG motif at the start of the activation loop points into the ATP binding site. Type I inhibitors are ATP-competitive and represent the majority of currently approved kinase inhibitors. Type II inhibitors bind to the inactive DFG-out conformation, wherein the Asp of the DFG motif flips by approximately 180° relative to the active conformation, resulting in Asp and Phe swapping their positions. Type II inhibitors occupy a characteristic hydrophobic pocket adjacent to the ATP binding pocket, which is only accessible in the DFG-out conformation. Type II inhibitors can be either ATP-competitive or not, depending on whether they extend past the gatekeeper into the adenine pocket and form hydrogen bonds with the hinge residues. Type III and Type IV inhibitors are not ATP-competitive. Type III inhibitors bind to an allosteric pocket opposite the ATP binding pocket and is also known to induce conformational changes in the activation loop, forcing the αC helix to adopt an inactive conformation. They do not form any hydrogen bonding interaction with the hinge residues. Type IV inhibitors bind to any allosteric sites distant from the ATP binding pocket and induce conformational changes that render the kinase inactive.

The serendipitous discovery of imatinib binding to the ABL DFG-out conformation has spurred great interest in the development of type II inhibitors targeting the inactive kinase conformation [9,10,11]. One underlying view was that type II inhibitors are intrinsically more selective than type I inhibitors based on the observation that the residues surrounding the hydrophobic pocket exposed in the DFG-out conformation are not as highly conserved as those in the ATP binding pocket [4]. In addition, it was originally thought that many kinases are unable to adopt the inactive DFG-out conformation [4]. However, this view was challenged by Zhao et al. who demonstrated that 220 kinases can be targeted with a small library of 36 type II inhibitors [7]. Nevertheless, Vijayan et al. had recently obtained profiling results for structurally validated type II inhibitors identified through conformational analysis and reached the conclusion that type II inhibitors are statistically significantly (*p* < 10^−4^) more selective than type I inhibitors [6].

It has been established that the RAS/RAF/MEK/ERK mitogen-activated protein kinase (MAPK) signaling pathway is essential to cellular growth and survival [12]. Constitutive activation resulting from mutations in this pathway impacts approximately one-third of human cancers [13]. BRAF (V-RAF murine sarcoma viral oncogene homologue B1) is a serine/threonine kinase that functions in this pathway as a downstream effector of RAS. Its mutant BRAF V600E has proven to be a highly tractable target in this cascade for cancer therapy [14]. FDA has already approved four BRAF V600E inhibitors, namely, vemurafenib (Zelboraf, 2011) [15], dabrafenib (Tafinlar, 2013) [16], sorafenib (Nexavar, 2005) [17], and regorafenib (Stivarga, 2012) [18]. Vemurafenib and dabrafenib are type I inhibitors, while sorafenib and regorafenib are type II inhibitors.

Although there are still controversies regarding the relative merits of type I and type II kinase inhibitors, the fact is that launched products are heavily biased toward type I inhibitors. This is the reason why we choose to target DFG-out conformation in our effort to discover lead for BRAF V600E inhibition. We believe that type II inhibitors research is still a vibrantly developing and highly rewarding field for kinase drug discovery [19,20].

## 2. Results and Discussion

**Figure 1:**
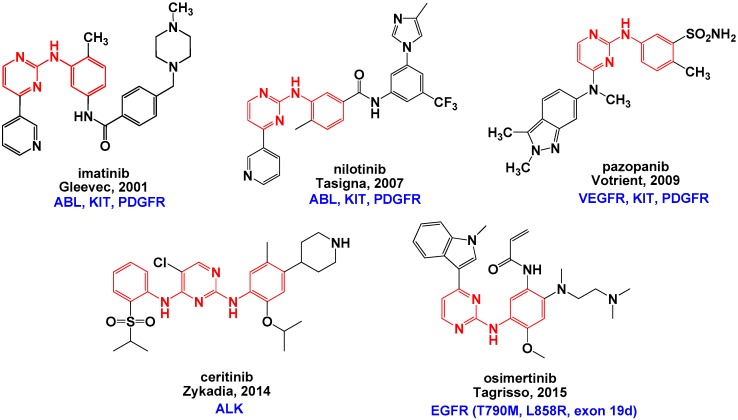
FDA-approved kinase inhibitors containing phenylaminopyrimidine (PAP). The PAP fragments are colored red. INN, brand name, year FDA approved, and main target kinases are provided for each kinase drug. Kinase abbreviations: ABL: Abelson kinase; KIT: stem cell factor receptor; PDGFR: platelet derived growth factor receptor; VEGFR: vascular endothelial growth factor receptor; ALK: anaplastic lymphoma kinase; EGFR: epidermal growth factor receptor.

**Figure 2:**
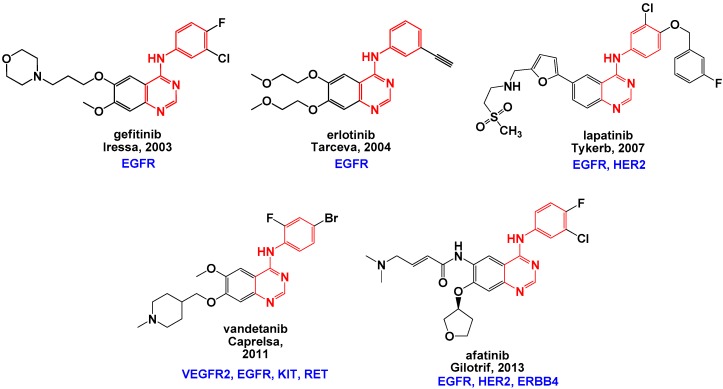
FDA-approved kinase inhibitors containing 4-anilinoquinazoline. The contained PAP fragments are colored red. INN, brand name, year FDA approved, and main target kinases are provided for each kinase drug. Kinase abbreviations: HER2 (ERRB2): erythroblastic leukemia viral oncogene homolog 2; RET: rearranged during transfection; ERRB4: erythroblastic leukemia viral oncogene homolog 4.

**Figure 3:**
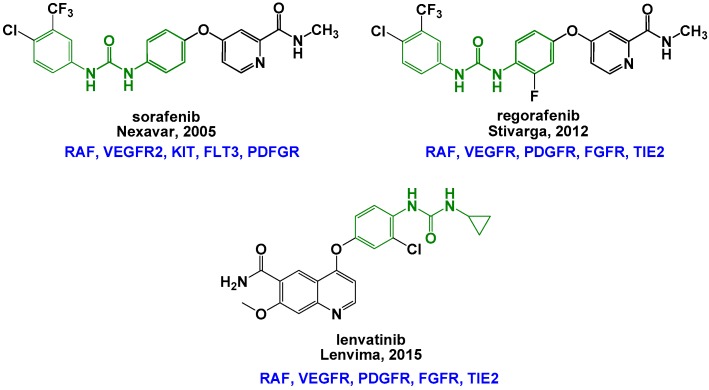
FDA-approved kinase inhibitors containing unsymmetrically disubstituted urea. The urea fragments are colored green. INN, brand name, year FDA approved, and main target kinases are provided for each kinase drug. Kinase abbreviations: RAF: rapidly growing fibrosarcoma; FLT3: Fms-like tyrosine kinase 3; FGFR: fibroblast growth factor receptor; TIE2: tyrosine kinase with immunoglobulin and epidermal growth factor homology domains 2.

Phenylaminopyrimidine (PAP), 4-anilinoquinazoline, and unsymmetrically disubstituted urea are identified as fragments that are frequently presented in 30 FDA-approved small molecule protein kinase inhibitors. PAP is presented in five (17%) launched protein kinase inhibitors (imatinib, nilotinib, pazopanib, ceritinib, and osimertinib) (Figure 1), 4-anilinoquinazoline is presented in five launched products (17%) (gefitinib, erlotinib, lapatinib, vandetanib, and afatinib) (Figure 2), while unsymmetrically disubstituted urea is presented in three launched products (10%) (sorafenib, regorafenib, and lenvatinib) (Figure 3). It is noteworthy that 4-anilinoquinazoline contains PAP in its skeleton, with PAP presented in 34% of approved protein kinase inhibitors. We therefore defined PAP and unsymmetrically disubstituted urea as privileged fragments for kinase drug discovery.

Thus, we were prompted to design type II BRAF V600E inhibitors based upon privileged fragments of PAP and unsymmetrically disubstituted urea. Sorafenib was employed as the template type II inhibitor which traps the structurally validated classical DFG-out conformation [6] of BRAF V600E (PDB code 1UWJ) [21].

**Figure 4:**
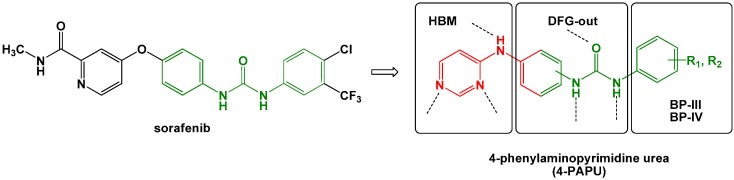
Evolution of type II kinase inhibitor pharmacophore 4-PAPU based upon privileged fragments of PAP (colored red) and unsymmetrically disubstituted urea (colored green). Potential hydrogen bond interactions are depicted as dashed lines.

**Figure 5:**
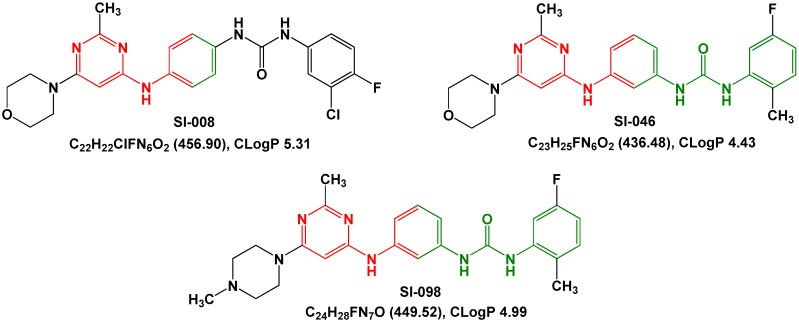
Selected compounds from the focused library based upon the pharmacophore 4-PAPU. PAP is colored red and unsymmetrically disubstituted urea is colored green. Molecular formula, molecular weight, and CLogP are generated with ChemDraw.

After changing the O linkage to NH and displacing 2-carboxamidopyridinyl with 4-pyrimidinyl, we traveled from sorafenib to a scaffold that fuses the two privileged fragments into one pharmacophore, which we coined 4-phenylaminopyrimidine urea (4-PAPU) (Figure 4). Importantly, the 4-PAPU scaffold fits the generalized pharmacophore model of type II inhibitors: the 4-pyrimidinyl hinge-binding moiety (HBM) is connected through a nitrogen atom to the central phenyl that is expected to occupy the DFG-out pocket (BPII). The urea functionality not only links the central phenyl and the terminal phenyl, but also serves as the hydrogen bond donor/acceptor to interact with the conserved glutamic acid in the αC helix and aspartic acid in the DFG motif. Substitutes R_1_ and R_2_ on the terminal phenyl occupy the lipophilic pockets created by the DFG-out flip (BPIII and BPIV) [22,23]. In our preliminary SAR campaign, a focused compound library based upon the pharmacophore 4-PAPU was synthesized and tested in a biochemical assay. It was found that the lead compound **SI-046** (IC_50_ 298 nM) exhibits BRAF V600E inhibitory activity comparable to the template compound sorafenib (IC_50_ 263 nM). **SI-008** (IC_50_ 685 nM) is moderately active, while **SI-098** (IC_50_ > 10,000 nM) is devoid of activity (Figure 5) (Table 1). The GlideScores of selected compounds generated with Glide, version 6.9 (Schrödinger, LLC, New York, NY, USA, 2015), which agrees with the biochemical results, are also presented in Table 1.

**Figure 6:**
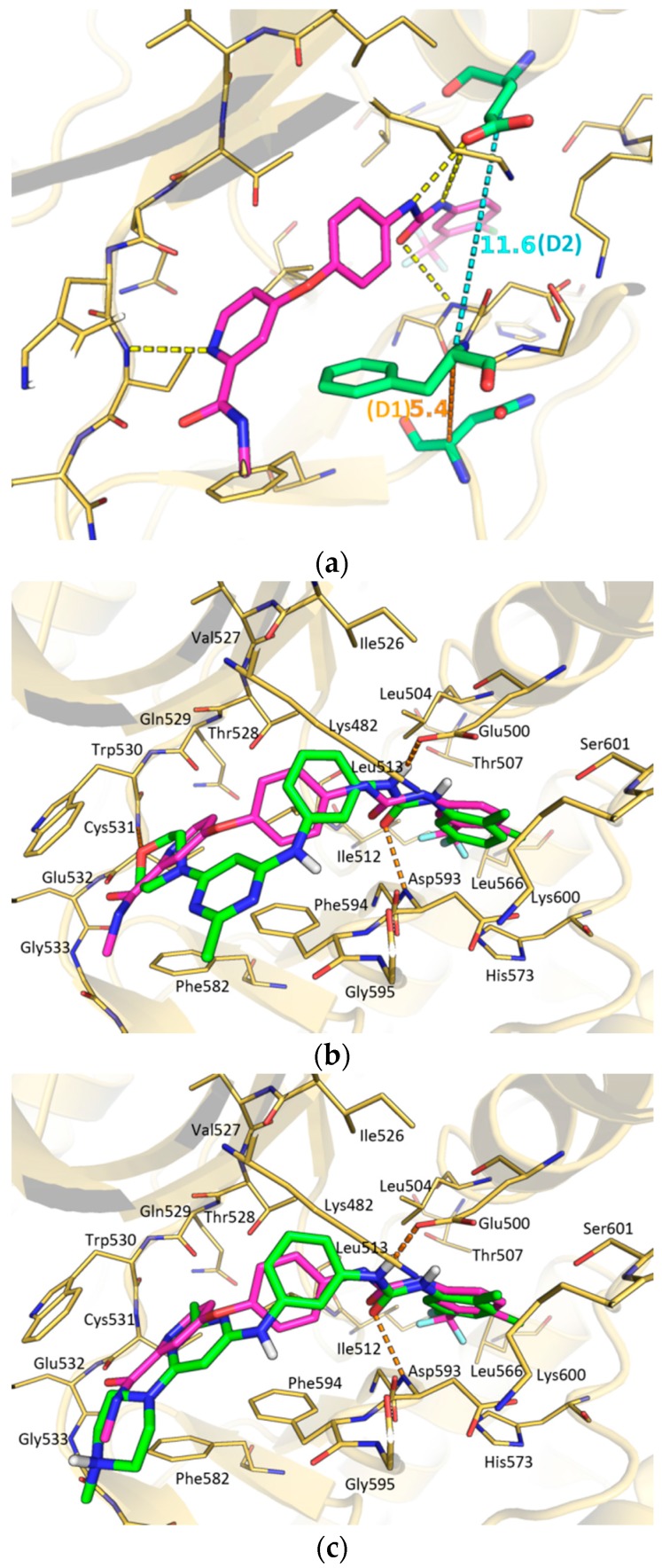
(**a**) The distance criteria D1 (5.4 Å) and D2 (11.6 Å) confirmed that sorafenib binds to the classical DFG-out conformation of BRAF-V600E (PDB code 1UWJ). Docking of (**b**) **SI-046** and (**c**) **SI-098** in the crystal structure of sorafenib bound to the classical DFG-out conformation of BRAF-V600E (PDB code 1UWJ). Sorafenib is colored magenta. **SI-046** and **SI-098** are colored green. H-bonds are depicted as orange dashed lines. Key interacting residues are illustrated in golden sticks.

Vijayan et al. [6] labeled those conformations for which D1 < 7.2 Å and D2 > 9 Å as “classical DFG-out” conformations. Those conformations that do not satisfy their structural definition of “classical DFG-out” were labeled “nonclassical DFG-out,” which cannot accommodate a type II inhibitor. D1 is the DFG Phe to Asn at HRD + 5 distance, while D2 is the DFG Phe to salt-bridge Glu distance. We measured the distance criteria D1 (5.4 Å) and D2 (11.6 Å) and confirmed that sorafenib binds to the classical DFG-out conformation of BRAF-V600E (PDB code 1UWJ) (Figure 6a). This agrees with the literature [6]. Molecular modeling was performed to establish the binding mode of selected title compounds. Docking of **SI-046** in the above-mentioned classical DFG-out conformation of BRAF V600E bound to sorafenib is depicted in Figure 6b. **SI-046** was shown to be bound to the DFG-out conformation of BRAF V600E. Its morpholino group binds in the hydrophobic pocket surrounded by Trp530, Cys531, and Phe582 and forms an H-bond with the backbone amide NH of Cys531 in the hinge region. The meta-disubstituted phenyl linking 2-methylpyrimidine ring through NH occupies the hydrophobic pocket of the ATP binding site and interacts with the side chain of Lys482 through a cation-p interaction. The side chain of Trp530 forms favorable p–p interaction (face-to-face) with 2-methylpyrimidine and p–p interaction (face-to-edge) with meta-disubstituted phenyl. Carbonyl and two NHs of urea form H-bond interactions with the backbone of Asp593 and the side chain of Glu500, respectively. Terminal 2-methyl-5-fluorophenyl extends into the hydrophobic pocket produced by the flip of Phe594 and lined with Leu504, Thr507, Leu566, Ile512, lys600, and His573. However, when the morpholino is displaced with 4-methylpiperazin-1-yl (**SI-098**), the interaction with the hinge region disappears due to clashes caused by the positively charged nitrogen on the piperazine ring (Figure 6c). Moreover, the higher absolute value of GlideScore of **SI-046** compared with **SI-098** indicated that **SI-046** forms more ideal interactions with the structurally validated DFG-out conformation of BRAF V600E. This agrees well with the experimental results (Table 1).

In summary, our approach extracted two privileged fragments—phenylaminopyrimidine (PAP) and unsymmetrically disubstituted urea—from FDA-approved small molecule protein kinase inhibitors and assembled them into 4-PAPU, a pharmacophore for type II inhibitors. Lead compound **SI-046**, which exhibited BRAF V600E inhibitory activity comparable to the template compound sorafenib in the biochemical assay, was in turn identified through preliminary SAR study. Docking data are consistent with the hypothesis that **SI-046** targets the classical DFG-out conformation of the oncogenic mutant BRAF V600E.

## 3. Materials and Methods

### 3.1. Chemistry

**Scheme 1:**
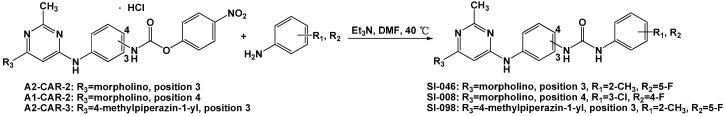
Synthesis of title compounds **SI-046**, **SI-008** and **SI-098**.

The title compounds **SI-046**, **SI-008**, and **SI-098** were prepared via the synthetic route outlined in Scheme 1 according to known procedures [24,25].

Melting points were determined on a Tianjing YRT-3 melting point apparatus (Tianda Tianfa Technology Co., Ltd., Tianjin, China) and were uncorrected. ^1^H-NMR spectra were recorded on a Varian INOVA-400 spectrometer (Varian, Inc., Palo Alto, CA, USA) at 400 MHz. Chemical shifts (δ) are reported in ppm relative to the DMSO-*d*_6_ signal (^1^H 2.50 ppm). Mass spectra were taken on a Waters Micromass Q-Tof micro instrument, and ionization was positive ion electrospray (MS-ESI) (Waters Corporation, Milford, MA, USA). Elemental analyses were performed on a Thermo SCIENTIFIC FLASH 2000 Organic Elemental Analyzer (Thermo Fisher Scientific Inc., New York, NY, USA).

*1-(5-Fluoro-2-methylphenyl)-3-(3-(2-methyl-6-morpholinopyrimidin-4-ylamino)phenyl)urea* (**SI-046**)

A mixture of **A2-CAR-2** [24] (0.73 g, 1.5 mmol), 5-fluoro-2-methylaniline (0.22 g, 1.76 mmol), and triethylamine (0.46 g, 4.5 mmol) in anhydrous DMF (6 mL) under nitrogen was stirred at 40 °C for 9 h. The resulting reaction mixture was diluted with methylene chloride (90 mL), then washed consecutively with aqueous 0.5 mol/L sodium hydroxide (2 × 25 mL) and water (2 × 25 mL), dried over anhydrous sodium sulfate, and evaporated in vacuo. The obtained residue was subjected to silica gel column chromatography eluting with a gradient of ethanol in ethyl acetate (0%–16%, *v*/*v*) to deliver **SI-046** as a white solid (0.23g, 35% yield): m.p. 220.5–221.5 °C; ^1^H-NMR (DMSO-*d*_6_) δ 8.93 (s, 1H, exchangeable), 8.70 (s, 1H, exchangeable), 8.59 (s, 1H, exchangeable), 7.73 (t, *J* = 2.0 Hz, 1H), 7.43 (dd, *J* = 2.0, 12.0 Hz, 1H), 7.13–7.17 (m, 3H), 7.00 (dd, *J* = 2.4, 8.0 Hz,1H), 6.95–6.97 (m, 1H), 5.95 (s, 1H), 3.66–3.68 (m, 4H), 3.46–3.48 (m, 4H), 2.31 (s, 3H), 2.16 (s, 3H); MS-ESI (*m*/*z*) 437.17 [M + H]^+^, 873.40 [2M + H]^+^; elemental analysis (C_23_H_25_FN_6_O_2_·0.25H_2_O) C, H, N, calcd. 62.64, 5.83, 19.06; found 62.62, 5.86, 18.84.

*1-(3-Chloro-4-fluorophenyl)-3-(4-(2-methyl-6-morpholinopyrimidin-4-ylamino)phenyl)urea* (**SI-008**)

This compound was prepared from **A1-CAR-2** [26] (0.49 g, 1.0 mmol) and 3-chloro-4-fluoro aniline (0.16 g, 1.1 mmol) in essentially the same way as that of **SI-046** to deliver **SI-008** as pale yellow crystalline powders (0.32g, 70% yield): m.p. 228.6–232.6 °C; ^1^H-NMR (DMSO-*d*_6_) δ 8.77 (s, 1H, exchangeable), 8.75 (s, 1H, exchangeable), 8.54 (s, 1H, exchangeable), 7.74 (d, *J* = 6.8 Hz, 1H), 7.29–7.45 (m, 6H), 5.73 (s, 1H), 3.65 (m, 4H), 3.42 (m, 4H), 2.29 (s, 3H); MS-ESI (*m*/*z*) 457.14 [M + H]^+^, 913.43 [2M + H]^+^; elemental analysis (C_22_H_22_ClFN_6_O_2_) C, H, N, calcd. 57.83, 4.85, 18.39, found 58.12, 5.16, 19.00.

*1-(5-Fluoro-2-methylphenyl)-3-(3-(2-methyl-6-(4-methylpiperazin-1-yl)pyrimidin-4-ylamino)phenyl)urea* (**SI-098**)

This compound was prepared from **A2-CAR-3** [24] (0.75 g, 1.5 mmol) and 5-fluoro-2-methylaniline (0.22 g, 1.76 mmol) in essentially the same way as that of **SI-046** to deliver **SI-098** as pale yellow crystalline powders (0.53g, 79% yield): m.p. 177.8–179.1 °C; ^1^H-NMR (DMSO-*d*_6_) δ 8.88 (s, 1H, exchangeable), 8.71 (s, 1H, exchangeable), 8.59 (s, 1H, exchangeable), 7.75 (s, 1H), 7.45 (d, *J* = 12.0 Hz, 1H), 7.16 (m, 3H), 7.01 (s, 1H), 6.95 (s, 1H), 5.97 (s, 1H), 3.50 (m, 4H), 2.37 (m, 4H), 2.31 (s, 3H), 2.22 (s, 3H), 2.17 (s, 3H); MS-ESI (*m*/*z*) 450.06 [M + H]^+^, 899.14 [2M + H]^+^; elemental analysis (C_24_H_28_FN_7_O) C, H, N, calcd. 64.12, 6.28, 21.81, found 63.72, 6.28, 21.55.

### 3.2. Biochemical Assays

A LanthaScreen kinase assay (Invitrogen) was used to measure the potency of title compounds against BRAF V600E. Shanghai ChemPartner Co., Ltd. (Pudong, Shanghai 201203, China) generated the kinase inhibitory data.

### 3.3. Docking

All of the modeling calculations in this work were performed using Glide, version 6.9 (Schrödinger, LLC, 2015) [27,28,29]. Before docking was carried out, the OPLS3 (Schrödinger, LLC, 2013) force field [30,31] was used to model the ligand and protein, and their charges were assigned using the Prime, version 4.2 (Schrödinger, LLC, 2015). The X-ray cocrystal structure of BRAF V600E (PDB code 1UWJ) was downloaded from RCSB Protein Data Bank. Water molecules and small inorganic ions were removed. Default parameters in Glide 6.9 were used in this study. The ligand poses that Glide generated passed through a series of hierarchical filters that evaluated the ligand’s interaction with the target. The initial filters tested the spatial fit of the ligand to the defined active site. Poses that passed these initial screens entered the final stage of energy minimization.

The Schrödinger’s proprietary GlideScore multi-ligand scoring function was used to score the poses:

GScore = 0.05 × vdW + 0.15 × Coul + Lipo + Hbond + Metal + Rewards + RotB + Site(1)

where GScore stands for GlideScore, vdW stands for Van der Waals energy, Coul stands for Coulomb energy, Lipo stands for Lipophilic term, HBond stands for Hydrogen-bonding term, Metal stands for Metal-binding term, Rewards stands for various features such as buried polar groups, hydrophobic enclosure, correlated hydrogen bonds, amide twists, and so on, RotB stands for Penalty for freezing rotatable bonds, and Site stands for Polar interactions in the active site.

The best poses were selected for representation. This score is intended to be more suitable for comparing the binding affinities of different ligands than is the “raw” Coulomb-van der Waals interaction energy. GlideScore shows exceptionally high accuracy in ranking the binding modes of the ligands.

## 4. Conclusions

Our privileged fragments-based approach was shown to efficiently deliver a bona fide type II kinase inhibitor lead targeting the structurally validated DFG-out conformation. Furthermore, the theme of this article is to showcase the strategy and rationale of our approach. Additional work will be required to enhance the potency and assess the selectivity of these molecules to help answer the question of whether type II inhibitors are more selective than type I. Finally, novel privileged fragments and assembling strategies should be innovatively pursued to explore diversified and emerging kinase targets.

## Figures and Tables

**Table 1:** BRAF V600E inhibitory activity and GlideScore of selected compounds from the focused library based upon the pharmacophore 4-PAPU.

Compound	IC_50_ (nM)	GlideScore (kJ/mol)
**SI-046**	298	11.3
**SI-008**	685	10.3
**SI-098**	>10,000	10.2
**sorafenib**	263	12.1

## Abbreviations

The following abbreviations are used in this manuscript:

ATP	adenosine triphosphate
DFG	Asp-Phe-Gly
FDA	Food and Drug Administration
HRD	His-Arg-Asp
INN	International Nonproprietary Names
PDB	Protein Data Bank
SAR	structure-activity relationship
